# Diagnostic accuracy of the neurological upper limb examination I: Inter-rater reproducibility of selected findings and patterns

**DOI:** 10.1186/1471-2377-6-8

**Published:** 2006-02-16

**Authors:** Jorgen R Jepsen, Lise H Laursen, Carl-Goran Hagert, Svend Kreiner, Anders I Larsen

**Affiliations:** 1 Department of Occupational Medicine, Sydvestjysk Sygehus, Østergade 81–83, DK-6700 Esbjerg, Denmark; 2 Department of Orthopaedic Surgery, University Hospital, S-22185 Lund, Sweden; 3 Department of Biostatistics, University of Copenhagen, DK-2200 Copenhagen, Denmark; 4 Occupational Health Services, Novozymes, DK-2880 Bagsværd, Denmark

## Abstract

**Background:**

We have previously assessed the reproducibility of manual testing of the strength in 14 individual upper limb muscles in patients with or without upper limb complaints. This investigation aimed at additionally studying sensory disturbances, the mechanosensitivity of nerve trunks, and the occurrence of physical findings in patterns which may potentially reflect a peripheral neuropathy. The reproducibility of this part of the neurological examination has never been reported.

**Methods:**

Two blinded examiners performed a semi-quantitative assessment of 82 upper limbs (strength in 14 individual muscles, sensibility in 7 homonymous territories, and mechanosensitivity of nerves at 10 locations). Based on the topography of nerves and their muscular and cutaneous innervation we defined 10 neurological patterns each suggesting a focal neuropathy. The individual findings and patterns identified by the two examiners were compared.

**Results:**

Strength, sensibility to touch, pain and vibration, and mechanosensitivity were predominantly assessed with moderate to very good reproducibility (median κ-values 0.54, 0.69, 0.48, 0.58, and 0.53, respectively). The reproducibility of the defined patterns was fair to excellent (median correlation coefficient = 0.75) and the overall identification of limbs with/without pattern(s) was good (κ = 0.75).

**Conclusion:**

This first part of a study on diagnostic accuracy of a selective neurological examination has demonstrated a promising inter-rater reproducibility of individual neurological items and patterns. Generalization and clinical feasibility require further documentation: 1) Reproducibility in cohorts of other composition, 2) validity with comparison to currently applied standards, and 3) potential benefits that can be attained by the examination.

## Background

With a prevalence of approximately 20% of the general population [1] chronic upper limb pain and physical impairment constitute diagnostic challenges to clinicians in many specialties (family medicine, orthopaedic surgery, rheumatology, neurology, and occupational medicine etc.). Patients may be undiagnosed or labelled with non-specific diagnostic acronyms, e.g. RSI (repetition strain injury), because the physical examination often fails to identify well-described clinical conditions. Commonly associated symptoms such as weakness and paraesthesiae [2] suggest involvement of the peripheral nerves.

Muscle weakness of Grade 0, 1 and 2 as proposed by Seddon [3] is easily noticeable in terms of impaired active motion and abnormal limb posture. Classic adverse postures induced by muscle imbalance caused by anatomically strictly outlined pareses include the waiter's tip position (paretic spinati, deltoid, biceps, brachialis and supinator muscles from an upper trunk injury), drop hand (paretic wrist, thumb and finger extensors from a radial nerve injury at upper arm level), and "claw hand" (intrinsic muscle paresis from an ulnar nerve injury at the wrist). These examples illustrate the diagnostic potential of the identification of abnormal postures induced by characteristic patterns of muscle weakness. Minor weakness of the individual muscles, e.g. of Grade 4, however, are not immediately visible, but can be reliably identified by a careful manual evaluation and are related to the presence of symptoms [4]. Similar reasoning relates to other parts of the neurological examination which according to a general consensus should be included in the evaluation of patients presenting with upper limb pain, weakness, and/or numbness/tingling. While the neurological examination aims to identify patterns that may reflect a nerve affliction the actual capability to do so depends of the content and execution of the examination and its quantification. An insufficient examination may result in information of potential diagnostic assistance being missed.

As a part of the estimation of the diagnostic accuracy of the physical examination in a sample of patients with and without upper limb complaints, we have previously presented the reproducibility of manual assessment of muscle strength in selected individual muscles [4]. This study aimed to address the inter-rater reproducibility of sensibility examined at homonymously innervated territories, of mechanosensitivity of nerves at specific locations, and of the occurrence in patterns of weakness, sensory deviations from normal and focal mechanical allodynia of nerves. Even with widespread use of the neurological examination, the reproducibility of this critical part of the examination is unknown.

## Methods

### Participants

Consecutive patients with any disorder (upper limb, low back, lung, etc.) attending the Department of Occupational Medicine, Sydvestjysk Sygehus Esbjerg were considered for enrolment in the study. The department is a secondary referral centre for assessment of the work-relatedness of any disorder and consequences regarding work capacity.

**Figure 1:**
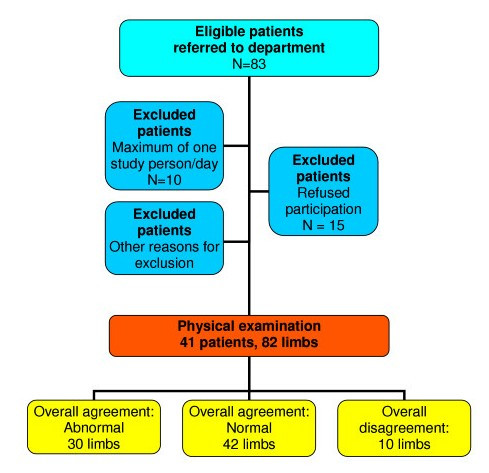
Flow diagram illustrating the patient sample and the main findings.

In order to secure instructions and blinding, patients were excluded when known to the examiners from earlier contacts with the department, when foreign language speaking or when presenting visible indication of disease, e.g. scars from prior upper limb surgery or an appearance suggesting recognizable disease such as an antalgic position. In addition, the sample was limited to the first eligible patient each day during the study. The study sample constituted 41 patients/82 limbs (Figure 1). Based on presuppositions with regard to the distribution of deviations from normal of the physical findings, this sample size was determined to be adequate to ensure statistical calculations of sufficient power. Data were collected prospectively.

The study complied with the Helsinki declaration. It was approved by the local Ethics Committee and signed informed consent was obtained from all participants.

### Physical examination and diagnostic interpretation

Two authors (JRJ and LHL) performed identical physical examinations comprising the parameters in Table 1. The examinations were performed in immediate succession one after the other and were based on simple measures and standard equipment. Both examiners were blinded to any information relating to the patients' history. Except for instructions from examiners and the patients' responses to the applied tests no communication occurred during the examinations.

#### Muscle strength

**Figure 2:**
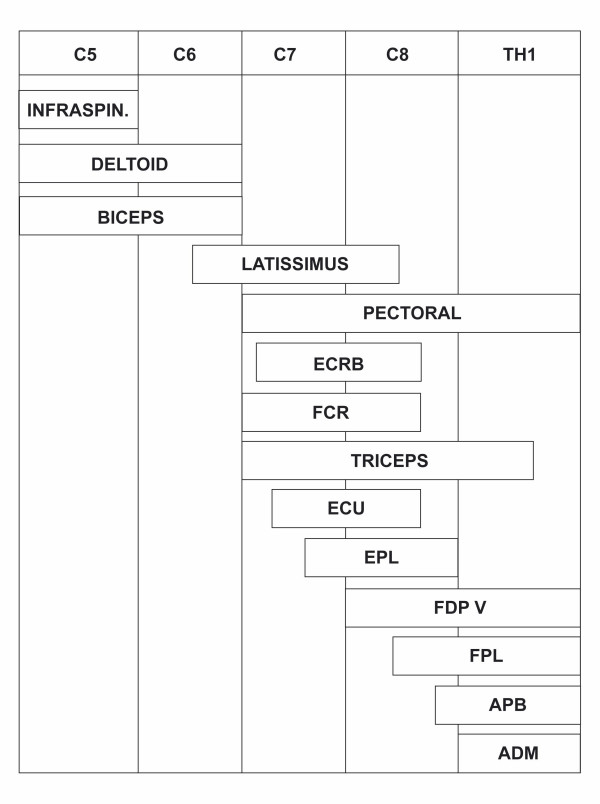
Distribution of 14 upper limb muscles in relation to the cervical roots.

The strength was evaluated individually in 14 muscles considered to be representative of the upper limb nerves (Table 2, Figure 2), using a technique designed by one of the authors (C-G H) and previously presented in details [4]. The manual examination was performed systematically from proximal to distal with consistent comparison right and left. The limb was positioned and stabilized in three different postures chosen to maximize the isolated action of each muscle studied (Table 2). Strength was quantified according to Table 1.

#### Sensibility

The sensibility to moving touch [5,6] and pinprick was examined in 7 homonymous innervated upper limb territories (Table 3). Perception of vibration was examined by a tuning fork 256 Hz [7] at the volar tips of the second and fifth fingers. Sensibility was quantified according to Table 1. Deviation of sensibility was classified as "marked" when an allodynic reaction was recorded, or when touch, pain or vibration could either not be perceived at all or was reduced sufficiently to be clearly apparent to the examiner from the patient's reaction. Deviation of sensibility was classified as "mild/any" with any other divergence from normal (hypo- or hypersensibility). For the latter assessment, findings were compared with sensibility in other territories assessed as normal.

#### Mechanosensitivity of nerve trunks

Nerves were palpated with a manual pressure of 3 kp from proximal to distal at 10 locations [8-11] (Table 4). Mechanical allodynia was quantified according to Table 1. "Marked" mechanical allodynia was registered with avoidance reaction/jump sign, "medium" allodynia when the patient expressed the pressure as seriously uncomfortable, and "mild/any" allodynia with the presence of any other soreness regarded as exceeding normal. For the latter assessment, the level of mechanical allodynia was compared to reactions regarded as normal to pressure elsewhere along nerves.

#### Dichotomization of the individual parameters

For the assessment of inter-rater reproducibility the scores were redefined for each individual muscle [4], sensory territory (Table 3), and localized mechanosensitivity (Table 4). Scores were recorded as abnormal when exceeding 0 (Table 1).

#### Definition of patterns and classification of limbs with respect to presence of patterns

**Figure 3:**
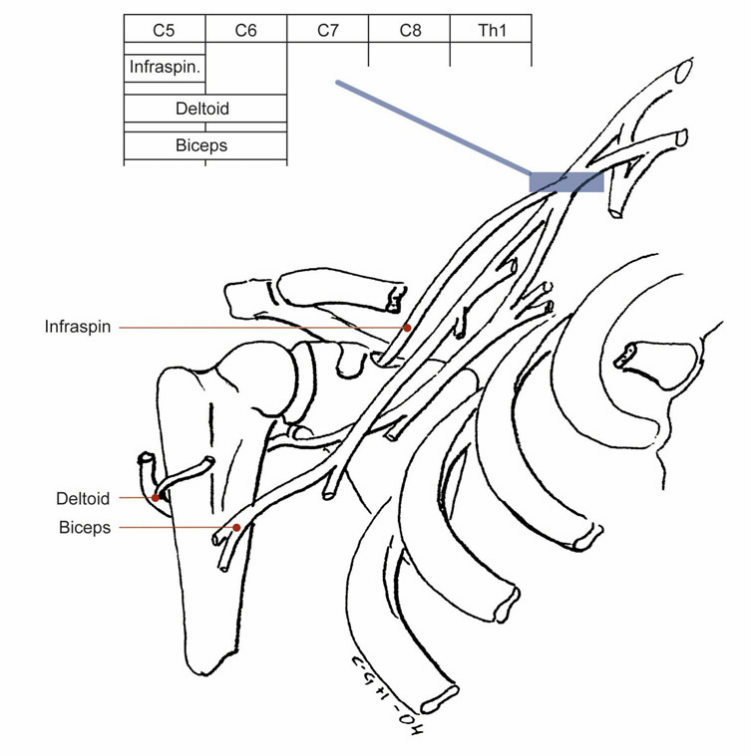
**Pattern of muscle weakness with upper trunk involvement. **Pareses of Infraspinatus, Deltoid and Biceps muscles. Note: The FCR muscle should always be found normal with an isolated upper trunk involvement

**Figure 4:**
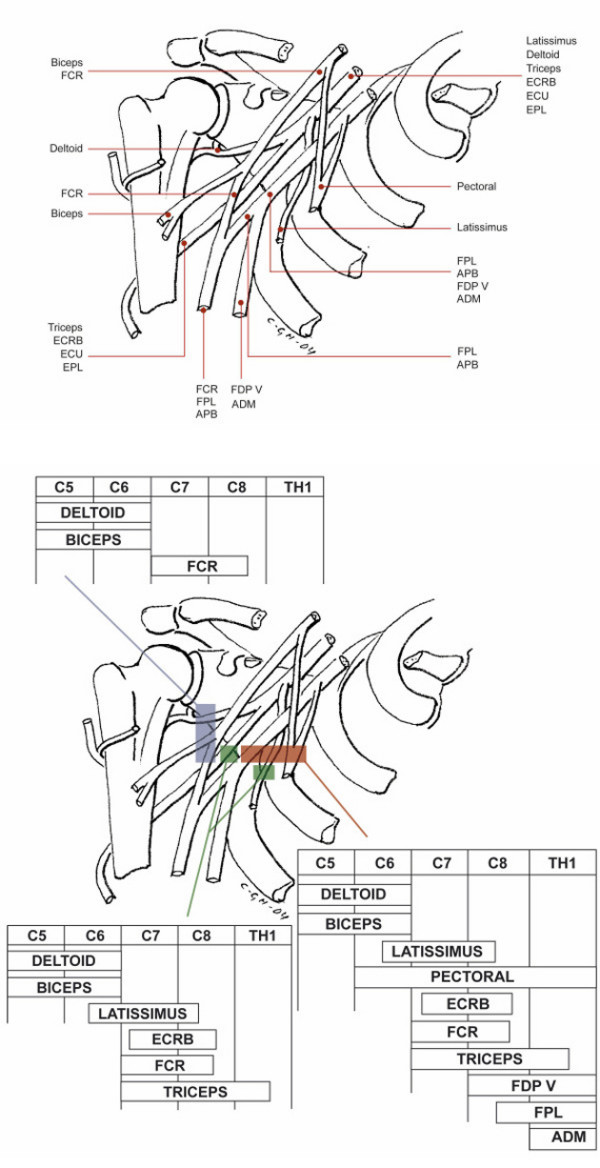
**The position of the nerves to selected muscles within the infraclavicular brachial plexus and the patterns of muscle weakness with an infraclavicular plexus involvement. **The involvement may be limited to its lateral part only (pareses of Deltoid, Biceps and FCR muscles), also include its intermediate part (in addition pareses of Latissimus, Triceps and ECRB muscles), or even its medial part (in addition to all the previous, pareses of Pectoral and ADM muscles). Note: The Infraspinatus muscle is normal with an isolated infraclavicular plexus involvement. For abbreviations see Table 2

**Figure 5:**
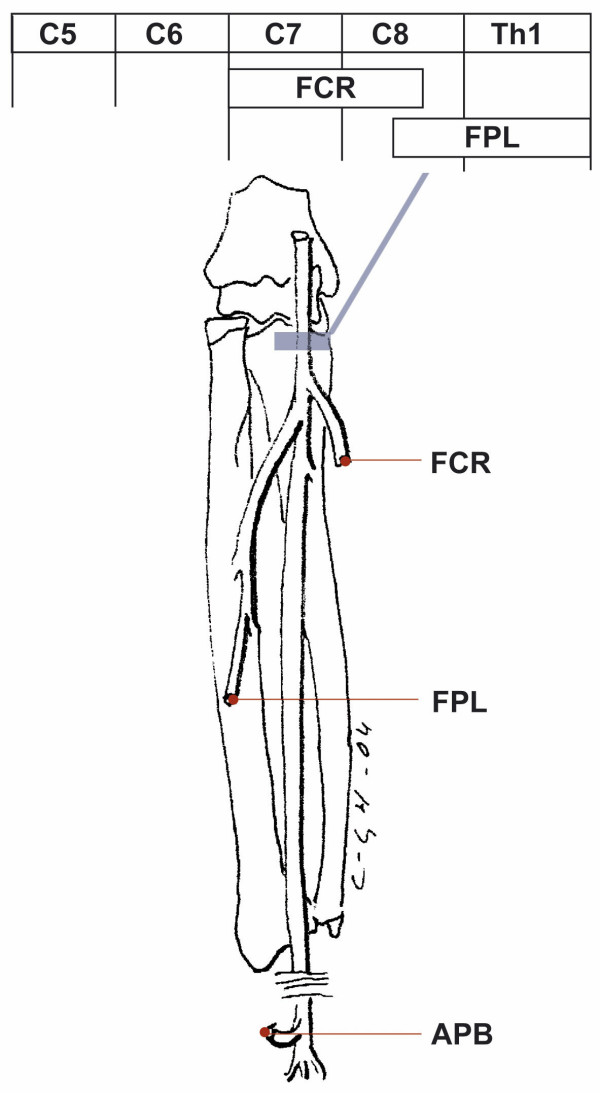
**Pattern of muscle weakness with medial nerve involvement. **Involvement at the elbow level, pronator syndrome causes pareses of FCR and FPL muscles. Involvement at the wrist level, carpal tunnel syndrome, causes paresis of the APB muscle. For abbreviations see Table 2

**Figure 6:**
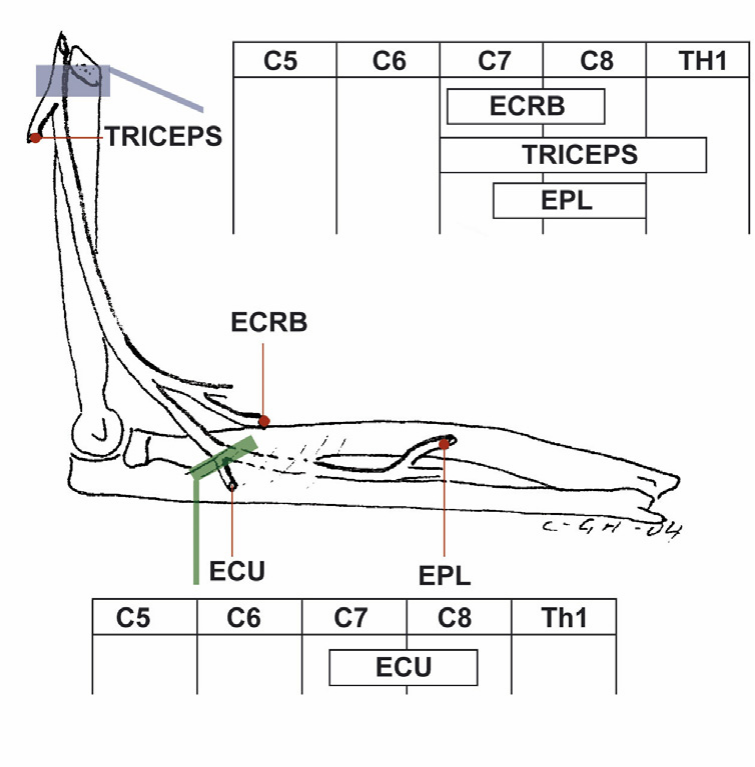
**Pattern of muscle weakness with radial and interosseous nerve involvement. **Radial nerve involvement at the upper arm level causes pareses of Triceps, ECRB and EPL muscles. Posterior interosseous nerve involvement at the level of the Supinator muscle, arcade of Frohse, radial tunnel syndrome causes paresis of the ECU muscle. For abbreviations see Table 2.

**Figure 7:**
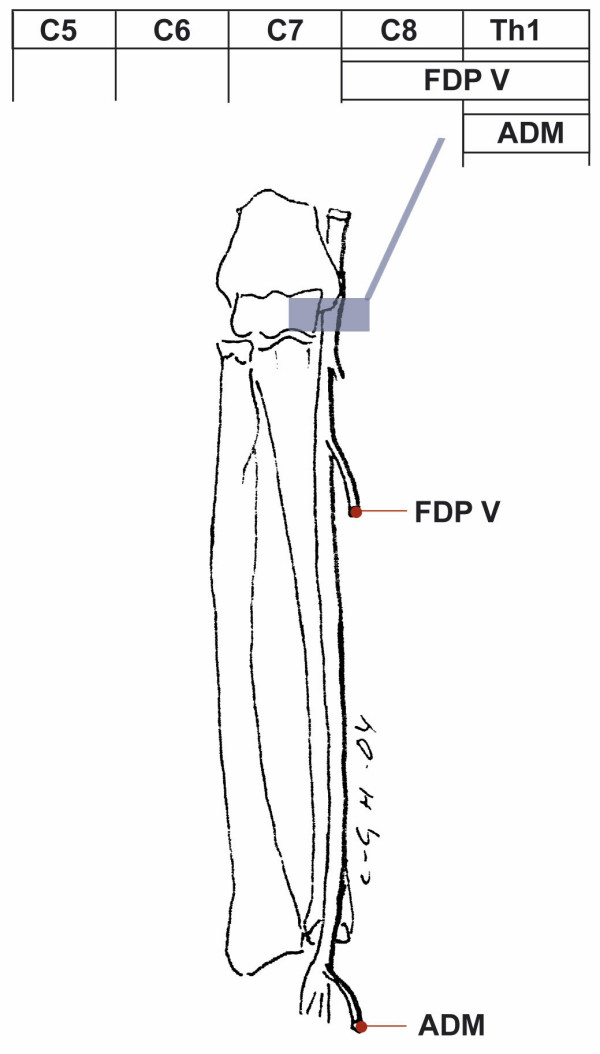
**Pattern of muscle weakness with ulnar nerve involvement. **Ulnar nerve involvement at the elbow level, cubital tunnel syndrome causes pareses of FDP V and ADM muscles, and at the wrist level, Guyon's canal, paresis of the ADM muscle. For abbreviations see Table 2

Based on the topography of each nerve and their motor and (for nerves with sensory afferents from the skin) sensory innervation, ten patterns of neurological findings were defined, each suggesting a specific location of nerve affliction (Table 5, Figures 3,4,5,6,7).

Each limb was classified with respect to the presence of one or several patterns (Table 5). This classification was based on the contribution of all applicable parameters with arbitrarily defined cut-off levels for scores for the individual items:

• For nerves without sensory afferent components from the skin (suprascapular and posterior interosseous nerves): A score of 1 or more for strength *and *mechanical allodynia *and *a score of 2 or more for at least one of the two (Table 1).

• For all remaining nerves (Table 5): A score of 1 or more for each of the three parameters strength, sensibility, *and *mechanosensitivity, but with a score for sensibility of 1, the score for strength *or *mechanosensitivity should be at least 2 (Table 1).

• The patterns were defined to reflect the most proximal location for which the criteria were met. A pattern reflecting a more distal affliction in the same nerve was additionally classified as present when the scores of the distal parameters were at least as high as the score of the corresponding proximal parameters (Table 1). E.g., with identification of a pattern reflecting the brachial plexus at cord level, a carpal tunnel pattern was additionally identified when the strength was reduced as much in the APB muscle as in the posterior deltoid, biceps and FCR muscles, *and *when mechanical allodynia over the carpal tunnel was at least as at the level of the infraclavicular brachial plexus (Tables 2, 4, 5).

### Statistics

#### Comparison of dichotomized data

Cohen's 6 statistics, a measure for testing whether agreement between raters of categorical data exceeds chance levels, was used for the analyses of the inter-rater variation of the dichotomized individual parameters and of the overall presence of any pattern: 6 = (p_o _- p_e_)/(1 - p_e_) where p_o _is the proportion of observed agreement and p_e _is the proportion of agreement expected by chance. The 6-coefficient has a maximum of 1.0 and is interpreted as 6: = 0.2 = poor, 0.21 – 0.40 = fair, 0.41 – 0.60 = moderate, 0.61 – 0.80 = good, 0.81 – 1.00 = very good [12].

#### Comparison of metrical data relating to the patterns

Dichotomous classification into the various patterns of physical findings may result in imperfect agreement, even with minor differences between the two raters (Table 5). For that reasons we have additionally examined the degrees of concordance between the examiners for each of the ten defined patterns. This has been achieved through construction of metrical scales from the addition of the scores for each of the three dimensions (strength, sensibility, and mechanosensitivity).

Whether or not these scales are continuous or defined by a fairly large set of discrete values, the evaluation of agreement was approached by dividing the problem of agreement into two different questions: 1) whether or not bias could influence rating in the sense that measurements of one rater are significantly larger or smaller than those of the other rater and 2) whether or not measurements by different raters are strongly correlated. These questions can be answered by paired t-tests and standard product-moment correlation coefficients which measure the degree of linear association between the two measurements. Agreement requires that responses to both questions are positive. A high degree of correlation, for instance, does not imply agreement unless measurements are unbiased.

A summary measure of degrees of association is a coefficient, measuring the degree of variance of differences between measurements that are explained by agreement. Such a measure for metrical scales can be defined in the following way: Let X_1 _and X_2 _be measurements by two different raters with D = X_1 _- X_2 _being the difference between the assessments of each rater. As a measure of degree of agreement we suggest the following ratio between the difference between the variance of D (assuming no agreement) and the observed variance of D divided by the variance of D (assuming no agreement), that is

VAR(X1)+VAR(X2)−VAR(D)VAR(X1)+VAR(X2).
 MathType@MTEF@5@5@+=feaafiart1ev1aaatCvAUfKttLearuWrP9MDH5MBPbIqV92AaeXatLxBI9gBaebbnrfifHhDYfgasaacH8akY=wiFfYdH8Gipec8Eeeu0xXdbba9frFj0=OqFfea0dXdd9vqai=hGuQ8kuc9pgc9s8qqaq=dirpe0xb9q8qiLsFr0=vr0=vr0dc8meaabaqaciaacaGaaeqabaqabeGadaaakeaadaWcaaqaaiabdAfawjabdgeabjabdkfasnaabmaabaGaemiwaG1aaSbaaSqaaiabigdaXaqabaaakiaawIcacaGLPaaacqGHRaWkcqWGwbGvcqWGbbqqcqWGsbGudaqadaqaaiabdIfaynaaBaaaleaacqaIYaGmaeqaaaGccaGLOaGaayzkaaGaeyOeI0IaemOvayLaemyqaeKaemOuaiLaeiikaGIaemiraqKaeiykaKcabaGaemOvayLaemyqaeKaemOuai1aaeWaaeaacqWGybawdaWgaaWcbaGaeGymaedabeaaaOGaayjkaiaawMcaaiabgUcaRiabdAfawjabdgeabjabdkfasnaabmaabaGaemiwaG1aaSbaaSqaaiabikdaYaqabaaakiaawIcacaGLPaaaaaGaeiOla4caaa@53D9@

One may argue that agreement is violated if the raters are biased in the sense that the distributions of measurements are different, and consequently that the degree of agreement should only be evaluated with no evidence of bias. We therefore suggest that the measure of agreement should be based on estimates of VAR(X_1_) and VAR(X_2_) assuming that both mean values and variances of the two sets of measurements are equal. The coefficient of agreement (λ) suggested above therefore is reduced to

λ=2⋅VAR(X)−VAR(D)2⋅VAR(X)
 MathType@MTEF@5@5@+=feaafiart1ev1aaatCvAUfKttLearuWrP9MDH5MBPbIqV92AaeXatLxBI9gBaebbnrfifHhDYfgasaacH8akY=wiFfYdH8Gipec8Eeeu0xXdbba9frFj0=OqFfea0dXdd9vqai=hGuQ8kuc9pgc9s8qqaq=dirpe0xb9q8qiLsFr0=vr0=vr0dc8meaabaqaciaacaGaaeqabaqabeGadaaakeaacqaH7oaBcqGH9aqpdaWcaaqaaiabikdaYiabgwSixlabdAfawjabdgeabjabdkfasjabcIcaOiabdIfayjabcMcaPiabgkHiTiabdAfawjabdgeabjabdkfasjabcIcaOiabdseaejabcMcaPaqaaiabikdaYiabgwSixlabdAfawjabdgeabjabdkfasjabcIcaOiabdIfayjabcMcaPaaaaaa@49BB@

with VAR(X) as the common estimate of the variance for each rater.

This measure of agreement for metrical scales is related to suggested methods [13] in which the difference between ratings and the variance of these differences is used as the natural starting point for the analysis of agreement. With the above mentioned assumptions that means and variances of ratings are exactly the same for the two raters, λ may be regarded as an estimate of the correlation coefficient and λ will mostly be fairly close to the sample correlation.

The correlation coefficient has been interpreted as λ: < 0.25 = little or no reliability, 0.25 ≤ λ < 0.50, fair, 0.50 ≤ λ < 0.75 = moderate to good, and λ ≥ 0.75 = good to excellent reliability [14].

### Role of the funding source

The funding sources have had no role in the study design, in the collection, analysis and interpretation of data, and in the decision to submit for publication.

## Results

### Participants

41 patients recruited between January 5^th ^and May 20^th ^1998 satisfied the inclusion criteria and participated in the index tests (Figure 1). 22 were males of median age 44 (range 29–61) years, and 19 females of median age 39 (range 25–52) years. Prior diagnostic difficulties, no responses to prior treatment or a recurrence of symptoms on resuming work were characteristics of most patients.

22 patients were referred due to complaints from one upper limb and 5 patients due to similar complaints from both upper limbs. Among patients referred for reasons other than upper limb complaints, 6 also had complaints pertaining to one of the upper limbs. Out of 44 non-symptomatic limbs, previous symptoms were reported in 15. Eight patients had never experienced upper limb symptoms.

No adverse events were observed from performing the index tests.

### Estimates of the inter-rater reproducibility

#### Individual physical findings

The reproducibility was moderate to good for most examined items. The previous assessment of individual muscle strength showed a median κ of 0.54 (0.25–0.72) [4]. For sensory qualities in terms of touch, pain, and perception of vibration, the median κ-values were 0.69 (0.31–0.90), 0.48 (0.42–0.69), and 0.58 (0.45–0.70), respectively (Table 3). Mechanical allodynia over the nerve trunks was assessed with a median κ of 0.53 (0.29–0.69) (Table 4).

#### Patterns of physical findings

With a median correlation coefficient of 0.75 (0.45–0.83), the ten patterns were identified with a fair to excellent reproducibility. The two examiners agreed on the presence of 90 patterns in 30 limbs meaning that patterns assigned to several locations were demonstrated in a high proportion of limbs (Tables 5 and 6).

With the applied definitions, the neurological involvement was assigned to the brachial plexus by the majority of the identified patterns. In all but one out of 21 instances in which the two examiners unanimously identified the pattern reflecting a brachial neuropathy at cord level, they additionally agreed on the presence of a distal pattern. The site of neurological involvement was assigned to the carpal tunnel in one and to the ulnar nerve at the elbow in two limbs (Table 5). In the absence of brachial plexus-involvement a pattern reflecting an individual nerve affliction was only unanimously recognized in few instances: Suprascapular nerve in three limbs, axillary nerve in one limb, and median nerve at elbow level in one limb. There was no unanimous identification of isolated root involvements or patterns assigned to afflictions of the musculocutaneous, radial, posterior interosseous, median (carpal tunnel), and ulnar (elbow level) nerves.

#### Identification of limbs with any defined pattern of physical findings

With a full consensus between the two examiners in 72 out of 82 limbs concerning the presence of any pattern in 30 limbs and the absence in 42 limbs, the overall inter-rater agreement of (42 + 30)/82 = 0.88 could be expressed as good with a κ-value of 0.75 (0.60–0.90) (Table 6).

## Discussion

The reproducibility for most dichotomized data (individual physical parameters and classification of limbs with respect to the presence of any defined pattern) was good and comparable and superior to that of other physical measures in common use, e.g., trigger point palpation [15], tendon reflexes [16] and for the lower limb the Babinski sign [17]. This result was achieved in spite of the innate weakness of the κ-statistics resulting in κ being reduced with a very high or low prevalence of the index condition even with excellent agreement (Tables 3, 4, 6). The reproducibility of manual muscle strength testing has resulted in recommendations for its clinical use [18]. It was still satisfactory after sub-classification of Seddon's Grade 4 [19] (Table 1) which is required to identify the minor strength-reductions characteristic to the sample under current study [4]. This study also confirms the reproducibility of sensibility testing shown by others [5]. While support for the diagnosis of nerve entrapment by the identification of tender nerves is acknowledged [20,21] we are unaware of previous studies relating to the reproducibility of this part of the examination.

The neurological upper limb examination is based on the recognition of specific patterns defined on the basis of anatomical facts relating to the nerve topography and muscular and cutaneous innervation. Each pattern aims to illustrate and locate a specific affliction of the nervous system. Taking into consideration the many patients for which the neurological examination is essential it is encouraging that good to excellent correlations between the two examiners were reached for eight out of ten defined patterns of mostly minor muscle weakness, sensory disturbances, and nerve tenderness. The correlation was no more than fair to moderate for patterns suggestive of upper trunk brachial plexopathy and suprascapular neuropathy which, however, were unanimously identified in a few instances only (Table 5).

Some of the findings may be unexpected. Patterns indicative of carpal tunnel syndrome and ulnar neuropathy at the elbow were rare in the studied sample. There was agreement in a limited number of limbs (five only) regarding the *isolated *occurrence of patterns reflecting distal afflictions but unanimously identified patterns in accordance with a brachial plexopathy were frequent (Table 5).

This study of the reproducibility of the neurological examination was conducted with its intended clinical application in mind. The presented formalized semi-quantitative examination is based on simple methods and equipment. It is logical and practical and can be used in any clinical setting. The reproducibility may be influenced by clinical variables such as the frequency and severity of the studied conditions in the sample.

The symptomatic patients referred for assessment in occupational medicine did not merely represent a group of chronic pain patients. While some patients presented with long-lasting and major disabling symptoms others have had minor symptoms for a short period of time. The duration of upper limb symptoms ranged from a few months to several years preceding referral. About half of the patients were on sick-leave while the remaining patients were able to continue their work. Most patients with upper limb symptoms were formerly diagnosed with specific disorders such as tennis elbow or shoulder tendonitis. Many had several such diagnoses suggested by various specialists. Others were labelled as non-specific upper limb conditions such as RSI (repetition strain injury). In many patients a neuropathic condition was suspected and electrophysiological studies (mostly of the median nerve in the carpal tunnel) and imaging (especially of the cervical spine) performed. These additional diagnostic studies did not contribute diagnostically. Previous treatment with NSAID, physiotherapy, surgery, etc. had been largely unsuccessful.

The sample-composition with 44 asymptomatic limbs and 38 symptomatic limbs variously affected on one or both sides represents a balanced distribution and a broad spectrum of disease. This was one advantage of the study and suggests the examination to be feasible in samples characterized by some variability in presentation and severity of upper limb disorders.

The expertise of the examiners is another crucial factor. Both have learned the techniques of examination rather recently. After two years of practice one of the examiners supervised the other in assessment of 20 patients before the study. In spite of independent performance and interpretation of the examination, misclassification into the defined patterns cannot be completely ruled out because all tests were performed by the same two examiners. The study design precludes the assessment of the magnitude of such potential bias.

## Conclusion

We have studied the reproducibility of a neurological upper limb examination consisting of an assessment of strength in representative muscles, sensory qualities in selected innervation territories and nerve trunk mechanosensitivity at defined locations. When applied to a sample of patients in occupational medicine the examination is reproducible in terms of individual physical findings and their occurrence in patterns.

Taking into account that only an estimated quarter of work-related upper limb disorders can currently be diagnostically classified by a standard physical examination [22], the frequent and reliable identification of neurological patterns in the studied sample suggests that a detailed formalized neurological examination may provide diagnostic assistance in a greater proportion of symptomatic limbs.

Generalization and clinical feasibility, however, demands further studies. The reproducibility should be studied in additional samples with different disease prevalence and severity. It is also essential that findings are accurate, i.e., that they reflect either a gold standard or other features of disorder. One example of construct validity is the relation of the identified patterns to the presence of upper limb symptoms. For the examination to be clinically feasible a beneficial effect of the examination on the course of disease or its prevention should also be demonstrated.

## Competing interests

The author(s) declare that they have no competing interests.

## Authors' contributions

C-G Hagert has developed the physical diagnostic approach concerning the systematic evaluation of strength in individual muscles and the identification of soreness at potential locations of neuropathy. In addition, professor Hagert is responsible for the hand-drawn figures. A I Larsen and J R Jepsen initiated and designed the study. A I Larsen, L H Laursen, and J R Jepsen collected the data. S Kreiner conducted the statistical analyses in cooperation with L H Laursen and J R Jepsen. L H Laursen, C-G Hagert, and J R Jepsen were responsible for the preparation of the manuscript.

## Pre-publication history

The pre-publication history for this paper can be accessed here:



## Figures and Tables

**Table 1:** Physical examination of the peripheral nerves by two blinded examiners (Scores in brackets)

**Manual testing of isometric strength in individual muscles**
Grading into five levels [3,19]:
• Grade 5 Contraction against powerful resistance, normal power (score = 0)
• Grade 4+ Contraction against gravity and strong resistance (score = 1)
• Grade 4 Contraction against gravity and moderate resistance (score = 2)
• Grade 4- Contraction against gravity and slight resistance (score = 3)
• Grade 3 * Contraction against gravity only (score = 3)
*No pareses minor than grade 3 were observed.

**Sensibility to light touch, pain (pinprick), and vibration (tuning fork 256 Hz)**

Grading into three levels:
• Normal (score = 0)
• Mild/any deviation of sensibility (score = 1)
• Marked deviation of sensibility (score = 2)

**Nerve trunks mechanosensitivity assessed by palpation**

Grading into four levels:
• No/normal tenderness (score = 0)
• Mild/any mechanical allodynia (score = 1)
• Medium mechanical allodynia (score = 2)
• Marked mechanical allodynia (score = 3)

**Table 2:** Postures employed for the examination of strength in 14 upper limb muscles

**Muscles**	**Patients' position at the physical examination**
Pectoralis major Posterior deltoid Latissimus dorsi	**I. **Both arms elevated in the shoulders to the horizontal plane, pointing straight forward with the elbows kept fully extended, neutral wrists and clenched fists.

Biceps Triceps Infraspinatus	**II. **Upper arms kept along the trunk, the elbows at 90° flexion and forearms directed forward, the wrists at neutral and the hands still in clenched fists.

	**III. **Leaning forward, the patient supported the forearms on the laps from the elbow to the wrist, the wrists free distal to the knees.

ECRB ECU	Forearms in pronation

EPL	Forearms in neutral

FPL FCR FDP V ADM APB	Forearms in supination

**Table 3:** Reproducibility of sensory testing in 82 limbs

**Sensory nerve**		**Number with agreement**								
		
		**Touch**			**Pain**			**Vibration**		
				
	**Innervated** ** territory**	**Nor- mal**	**Ab-nor-mal**	**κ-value (95% CI)**	**Nor- mal**	**Ab- nor-mal**	**κ-value (95% CI)**	**Nor- mal**	**Ab- nor-mal**	**κ-value (95% CI)**
Axillary	Deltoid region	68	8	0.69 (0.45–0.93)	52	15	0.54 (0.33–0.75)			
Medial cutaneous of arm	Medial upper arm	76	5	0.90 (0.71–1.00)	64	6	0.42 (0.14–0.69)			
Medial cutaneous of forearm	Medial forearm	74	5	0.75 (0.47–0.98)	65	10	0.69 (0.48–0.91)			
Musculocutaneous	Lateral forearm	71	6	0.67 (0.40–0.95)	56	11	0.48 (0.25–0.71)			
Radial	1^st ^dorsal web	69	3	0.31 (0.00–0.64)	56	11	0.48 (0.25–0.71)			
Median	Volar tip of index	69	8	0.73 (0.50–0.96)	54	11	0.43 (0.20–0.66)	41	29	0.70 (0.54–0.86)
Ulnar	Volar tip of 5^th ^digit	71	5	0.59 (0.29–0.89)	62	8	0.48 (0.23–0.74)	55	11	0.45 (0.22–0.68)

**Table 4:** Reproducibility of examination for mechanosensitivity of nerves in 82 limbs

**Nerve**	**Location**	**Number with agreement**		**κ-value (95% CI)**
			
		**No allodynia**	**Allodynia**	
Brachial plexus (upper trunk level)	Scalene triangle	51	14	0.48 (0.27–0.70)
Brachial plexus (cord level)	Infraclavicularly behind pectoralis minor muscle	30	37	0.63 (0.47–0.80)
Suprascapular	Suprascapular notch	39	15	0.29 (0.10–0.48)
Axillary	Quadrilateral space	33	29	0.52 (0.34–0.70)
Musculocutaneous	Passage through coracobrachial muscle	47	19	0.56 (0.37–0.75)
Radial/posterior	Upper arm (Triceps or brachioradialis arcades)	39	24	0.54 (0.37–0.71)
interosseous	Radiohumeral joint or supinator tunnel	27	30	0.41 (0.22–0.60)
Median	Elbow level	60	10	0.54 (0.32–0.77)
	Carpal tunnel	73	3	0.47 (0.10–0.83)
Ulnar	Elbow level	73	5	0.69 (0.40–0.98)

**Table 5:** Reproducibility of classification into defined patterns in 82 limbs (Abbreviations table 2)

**Location of mechanical allodynia**	**Patterns**		**Number of limbs classified in agreement**		**Correlation (95% CI)**
		
	**Reduced strength**	**Sensory deviations**	**Pattern absent**	**Pattern present**	
Brachial plexus (Upper trunk level), Figure 3	Infraspinatus, post. deltoid, biceps	Axillary, musculocutaneous	58	5	0.56 (0.39–0.69)
Brachial plexus (Cord level), Figure 4	Post. deltoid, biceps, FCR ^a^	Axillary, median, musculocutaneous	48	21	0.82 (0.73–0.88)
Suprascapular nerve (Suprascapular notch), Figure 3	Infraspinatus	-	55	4	0.45 (0.26–0.61)
Axillary nerve (Quadrilateral space), Figure 3-4	Posterior deltoid	Axillary	53	20	0.79 (0.69–0.86)
Musculocutaneous nerve (Coracobrachial muscle), Figure 3-4	Biceps	Musculocutaneous	66	4	0.74 (0.62–0.83)
Radial nerve (Upper arm), Figure 6	Triceps, ECRB, EPL	Radial	64	13	0.83 (0.75–0.89)
Posterior interosseous nerve, Figure 6	ECU	-	66	10	0.75 (0.64–0.83)
Median nerve (Elbow level), Figure 5	FCR, FPL	Median	54	10	0.82 (0.73–0.88)
Carpal tunnel, Figure 5	APB	Median	79	1	0.73 (0.61–0.82)
Ulnar nerve (Elbow level), Figure 7	FDP V, ADM	Ulnar	74	2	0.70 (0.57–0.80)

a. Depending of the extent of brachial plexus involvement, reduced strength in additional muscles were allowed. The infraspinatus muscle, however, should be intact (Figure 4).

**Table 6:** Classification into absence or presence of any of the defined patterns

		**Blinded examiner 2**		
			
		**Number of limbs without any pattern**	**Number of limbs with any pattern**	**Number of limbs**
	
**Blinded examiner 1**	**Number of limbs without any pattern**	**42**	6	48
	**Number of limbs with any pattern**	4	**30**	34
	
	**Number of limbs**	46	36	82

κ = 0.75 (95% CI: 0.60–0.90)
